# Outbreak of a beta-lactam resistant non-typeable *Haemophilus influenzae* sequence type 14 associated with severe clinical outcomes

**DOI:** 10.1186/s12879-015-1319-8

**Published:** 2015-12-23

**Authors:** Madelen Andersson, Fredrik Resman, Rickard Eitrem, Peter Drobni, Kristian Riesbeck, Gunnar Kahlmeter, Martin Sundqvist

**Affiliations:** Department of Infectious Diseases, Blekinge Hospital, Karlskrona, Sweden; Department of Translational Medicine, Medical Microbiology, Lund University, Malmö, Sweden; Department of Communicable Disease Control, County Blekinge Karlskrona, Sweden; Department of Clinical Microbiology, County Kronoberg Växjö/Karlskrona, Sweden; Department of Medical Sciences, Division of Clinical Bacteriology, Uppsala University, Uppsala, Sweden; Department of Laboratory Medicine, Clinical Microbiology, Faculty of Medicine and Health, Örebro University, Örebro, Sweden

**Keywords:** Ampicillin, Betalactamase negative, BLNAR, Long-term care facility, *Haemophilus influenzae*, Non-typeable, Outbreak, Resistant

## Abstract

**Background:**

During October 2011 several residents and staff members at a long-term care facility (LTCF) for elderly fell ill with respiratory symptoms. Several of the residents required hospitalization and one died. Non-typeable *Haemophilus influenzae* (NTHi) was identified as the causative pathogen.

**Methods:**

A descriptive analysis of the outbreak and countermeasures was performed. For each identified bacterial isolate implied in the outbreak, standard laboratory resistance testing was performed, as well as molecular typing and phylogenetic analysis.

**Results:**

The identified *H. influenzae* was beta-lactamase negative but had strikingly high MIC-values of ampicillin, cefuroxime and cefotaxime. All isolates displayed the same mutation in the *fts*I gene encoding penicillin-binding protein (PBP) 3, and all but one were identified as sequence type 14 by Multilocus Sequence Typing (MLST). In total 15 individuals in connection to the LTCF; 8 residents, 6 staff members and one partner to a staff member were colonized with the strain.

**Conclusion:**

This report illustrates the existence of non-typeable *H. influenzae* with high virulence, and furthermore emphasizes the importance of continuous surveillance of possible outbreaks in health care facilities and prompt measures when outbreaks occur.

## Background

*Haemophilus influenzae* is a respiratory tract commensal that is associated with upper respiratory tract infections, but also meningitis and septicemia [1]. After the introduction of a *H. influenzae* type b (Hib) vaccine in the childhood vaccination program in 1992, non-typeable *H. influenzae* (NTHi) have dominated *H. influenzae* morbidity in Sweden [2], but NTHi are less prone to cause severe infections than Hib in unvaccinated populations [3]. Recent studies have reported an increase in invasive disease caused by NTHi in individuals > 60 years of age [2] and a recent review has suggested increased surveillance and typing activities of NTHi [4]. Beta-lactam resistance in *H. influenzae* is caused either by the production of beta-lactamases or by amino acid substitutions in the penicillin binding protein (PBP) 3 resulting in decreased affinity for penicillins and cephalosporins [5]. The amino acid substitution pattern can be used to divide these isolates into three different groups [6]. Outbreaks of infections caused by NTHi in long-term care facilities (LTCF) for elderly are rare and have only been reported occasionally [7, 8].

### Outbreak description

On October 5^th^, 2011, three residents (all aged 80 years or older) from an LTCF were referred to the hospital of Karlskrona, Sweden, with fever, cough and respiratory distress. Due to the suspicion of an outbreak of a communicable respiratory tract pathogen, the general practitioner (GP) responsible for the LTCF contacted the County medical officer for Communicable Disease Control (CCDC). Patients were tested for influenza virus with polymerase chain reaction (PCR) and urinary antigen test for *Legionella pneumophila* serogroup 1. Samples from the nasopharynx, urine and blood were obtained for bacterial culture. All patients received benzylpenicillin (PcG) intravenously according to national guidelines. Due to suspicion of influenza, oseltamivir was administered to the hospitalized as well as the eight non-infected residents at the LTCF. Both influenza and Legionella tests were negative and oseltamivir was discontinued two days later.

The LTCF consisted of twelve separate apartments occupied by 11 residents. The staff generally wore protective clothing and had access to alcohol-based hand sanitizers. On October 5^th^, several staff members also had respiratory symptoms and a few were on sick-leave. Four staff members with mild respiratory tract symptoms consulted the Dept. of Infectious diseases on October 6^th^, encouraged by the CCDC, and were in addition to nasopharyngeal cultures sampled for influenza virus A and B (*n* = 3), *Mycoplasma pneumoniae* (*n* = 4) and Respiratory Syncytial virus (*n* = 3). Two of the hospitalized residents were diagnosed with pneumonia as confirmed by X-ray, while the third was considered as a bacterial bronchitis. On October 8^th^, nasopharyngeal cultures from all three hospitalized patients grew a beta-lactamase negative *H. influenzae* with positive screening for Beta-lactam non-susceptibility and resistant to co-trimoxazole (SXT). The isolates were susceptible to tetracycline (TET) and ciprofloxacin (CIP). The isolates were further characterized with MIC testing for ampicillin (AMP) and cefotaxime (CTX), and sent to the Swedish Institute for Communicable Disease Control (SMI) for capsular typing. On October 9^th^, a female resident without obvious symptoms of infection died at the LTCF. The autopsy showed that the cause of death was an extensive pneumonia, and an isolate obtained from the pulmonary tissue revealed a beta-lactamase negative Hi with an identical susceptibility pattern as the hospitalized residents. On October 10^th^, one more resident from the same LTCF was admitted to the hospital with pneumonia and was treated with doxycycline at admission. The nasopharyngeal culture yielded a Hi isolate with the same antibiogram as the previously hospitalized patients. A summary of the clinical information available for the four hospitalized residents is presented in Table 1.

**Table 1:** Clinical information of the hospitalized residents evaluated according to the CRB-65 score, plasma lactate and C-reactive protein (CRP) at admission

Resident	Gender	Resp. rate (min)	CRP (mg/L)	Saturation (%)	Temp. (°C)	Blood pressure (mmHg)	Lactate (mmol/L)	CRB-65 (score 0–4)	X-ray	Hospital stay (days)	Comorbidities
1	F	30	302	80	37,4	190/105	2.4	2	Cardiac incompensation, pleural fluid	5	Asthma
2	M	46	380	87	39,2	87/58	3.1	3	Infective infiltrates in both lungs	5	Hypothyreosis
3	F	28	269	81	37,4	130/60	2	1	Infective infiltrate in right lung	4	Asthma, Diabetes mellitus, Renal failure, Stroke, Vascular dementia
4	F	28	309	88	37,2	118/58	0.9	1	Infective infiltrates in the right lung	7	-

Several actions were taken by the CCDC three days after the initiation of the outbreak. Regular meetings were arranged with all responsible professionals according to the local epidemic plan. The hygiene routines at the LTCF were surveyed by the infection control team. It was agreed that there was an outbreak of an NTHi with an unusual susceptibility pattern, seemingly high virulence and ability to spread. No new residents were admitted to the LTCF until the spread was under control. Education was given regarding good hygienic routines and cohorting of staff was introduced.

## Methods

### Patient characteristics

A retrospective chart review was performed for the four hospitalized patients. Disease severity was evaluated using the CRB-65-system (confusion-respiratory rate-blood pressure-age >65 years) [9].

### Microbiological methods

Nasopharyngeal swabs (Copan, Brescia, Italy) were obtained from 60 individuals, as part of standard care: 10 residents (6 with/4 without symptoms), 49 staff members (7 with/42 without symptoms), and one relative to a staff member presenting with symptoms of upper respiratory tract infection. An additional sample was obtained from pulmonary tissue of the resident that died. Nasopharyngeal swabs were cultured according to standard methodology. In brief nasopharyngeal swabs were cultured on Blood agar incubated in anaerobic environment and Hematin agar incubated in 5 % CO_2_. Olandomycin-discs (50 μg) were used to improve the detection of *H. influenzae*. Species identification was performed using MALDI-TOF (Microflex and Biotyper 3.0, Bruker Daltonics, Billerica, MA) with standard parameters. Susceptibility testing was performed according to EUCAST.

In October 2011 all *H. influenzae* isolates isolated at the Dept. of Clinical Microbiology, Karlskrona were subjected to beta-lactam resistance screening using the PCG 1 unit disc as recommended by EUCAST. Briefly, isolates displaying a zone of PCG ≤ 12 mm were further analysed with Cefinase disk (Biomerieux, Solna, Sweden) to detect beta-lactamase production. Beta-lactamase negative isolates were further tested with Etest (Biomerieux) on MH-F agar to evaluate the susceptibility to AMP and CTX. In retrospect 14 isolates in this study were tested with Etest for susceptibility to cefuroxime (CXM). All isolates were also tested for susceptibility to TET, SXT, nalidixic acid (NAL), cefaclor (CKL), and ampicillin/clavulanic acid (ACL) using disk diffusion on MH-F agar according to EUCAST methodology (www.eucast.org). Finally, the total number of nasopharyngeal swabs analyzed during October 2009–2011 was retrieved from the Laboratory Information System.

### Molecular typing and phylogenetic analyses

The *fts*I gene was analysed as published by Skaare et al. [10]. Consensus and protein sequences were generated using BioEdit 7.0.9.0. The available outbreak isolates as well as 31 controls (21 invasive Swedish NTHi cases [11] and 10 randomly selected Swedish nasopharyngeal isolates were sequence-typed using PCR primers and conditions according to the *H. influenzae* protocol (http://haemophilus.mlst.net/). Sequences were trimmed manually, concatenated, aligned and edited using ClustalX [12]. A best-fitting nucleotide substitution model was estimated using jModeltest 0.1.9 [13]. A Maximum likelihood tree was constructed in MEGA5.05 [14] using the General Time Reversible model including invariable sites and rate variation (GTR + I + G). Support for internal branches was obtained by 1000 bootstrap replicates. The resulting phylogenetic tree was visualized using FigTree v.1.3.1 (http://tree.bio.ed.ac.uk/software/figtree).

### Ethics statement

The study was performed in accordance with approval by The regional ethic review board in Lund, Sweden (2014/533).

## Results

In total 15 individuals were positive for a beta-lactamase negative *H. influenzae* resistant to PCG and STX. Fourteen of these isolates were from nasopharynx (7 residents, 6 staff members, one partner to a staff member) and one from lung tissue (post-mortem culture). All isolates were susceptible to NAL and TET and displayed MIC values for AMP and CTX near the clinical breakpoints while CXM MIC was 16 mg/L. The MIC for AMP were 1 or 2 mg/L (current EUCAST breakpoints S ≤ 2 mg/L, R > 2 mg/L) and for CTX 0.125 or 0.25 mg/L (S ≤ 0125 mg/L, R > 0.125 mg/L). The reported result for ACL was inferred from the MIC-value from AMP. Isolates with this particular susceptibility pattern were not observed in any other clinical samples in October 2011 in County Blekinge. The three isolates sent to the Swedish CDC for capsule typing by PCR were determined as NTHi.

**Fig. 1:**
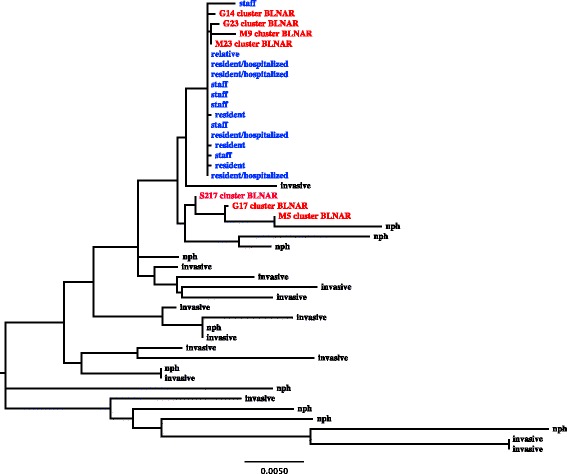
A phylogenetic tree from concatenated MLST sequences of the outbreak isolates (*n* = 14) as well as of 31 control isolates. The outbreak isolates are shown in blue text. Isolates from a suggested cluster of BLNAR genotype IIb isolates with identical PBP-3 sequences are shown in red (*n* = 7). Randomly selected controls are shown in black, where nasopharyngeal controls are denoted as “nph”, and invasive control are denoted as “invasive”. The tree shows a clonal clustering of the outbreak isolates, with one isolate separated from the rest. Interestingly, the outbreak isolates cluster with the suggested BLNAR clone [15]

Fourteen of the 15 NTHi isolates with the specific susceptibility pattern were saved and available for further analysis. Six isolates were from residents, 7 from staff members and one from a relative to a staff member. All isolates displayed the same *fts*l-gene sequence with a corresponding amino acid motif of Asn526Lys (genovar IIb [14]), and a homogenous MLST-pattern where all but one outbreak isolate were subtyped to be ST 14. Interestingly, the isolates clustered with invasive NTHi isolates from other parts of Sweden (Fig. 1).

In October 2011, 159 nasopharyngeal cultures in total were analyzed in County Blekinge. The corresponding figures for 2009 and 2010 were 94 and 113, respectively. The proportion of *H. influenzae* during the same 3-year period (2009–2011) was 7 %, 10 % and 13 %. The resistance to CTX has historically in this area been very low (single isolates per year) and in samples not related to the outbreak CTX-resistance was not observed during the time of the outbreak.

The first three residents admitted to hospital were administered PcG. All three experienced an initial clinical improvement. The fourth patient received doxycycline from start. All four patients admitted to the hospital and 9 of the colonized residents and staff, were treated with doxycycline. A control culture was obtained approximately three weeks later. The outbreak strain still colonized the nasopharyngeal flora in five of these individuals. None of the residents and staff required hospitalization due to respiratory tract infections during the weeks after the outbreak.

## Discussion

This outbreak demonstrates the potential virulence of subtypes of non-typeable *H. influenzae*, and highlights the important roles of all parts of the healthcare system to identify and control an outbreak. The outbreak was suspected by an alert GP, who contacted the CCDC leading to adequate sampling of the suspected index patients and quick measures of control. The detection was further facilitated by the new screening method for detection of beta-lactam resistance in *H. influenzae* recommended by EUCAST using PcG. The testing of AMP only had in these strains probably led to false conclusions, since AMP-resistance was not readily detected but cephalosporin resistance was pronounced. There were no geographically unrelated clinical isolates displaying the specific antibiogram identified during October 2011 in County Blekinge supporting the local characteristics of the outbreak.

A high attack rate was observed as 7 of the 11 residents were colonized, four of them were hospitalized and a fifth resident died due to pneumonia. In this patient no other pathogens than the specific Hi-strain were isolated from lung tissue. Several of the staff, without underlying disease, experienced respiratory tract symptoms at the time of the outbreak and in 6 of these the outbreak strain was isolated. The pathogenicity of NTHi is substantially lower and different than that of Hib [3], but in this outbreak an NTHi strain caused severe infections supporting the notion that some lineages of NTHi have a higher virulence. MLST and *fts*I-gene sequencing showed a clonal distribution of the epidemiologically related isolates, and the main ST-type was 14. Interestingly, the outbreak isolates clustered with a recently acknowledged lineage of invasive isolates of NTHI with the same *fts*I sequence from several larger regions in Sweden [11]. The ST 14 cluster has also been demonstrated as the subtype most prone to cause hospitalization among subtypes of NTHi in a recent Norwegian study [15]. *In vitro* studies have indicated that NTHi with specific changes in PBP3 may be more prone to invasion into the host epithelial cells through micropinocytosis [5], even though this is not likely to be related to the PBP-3 change per se [16]. It is unlikely that the isolation of the same *H. influenzae* in all cases would be coincidental, and that the outbreak was due to another microorganism. *Haemophilus influenzae* is not a common colonizer of the respiratory tract in healthy adults such as the staff [17], and a in a recent screening of 98 non-infected residents of a different Swedish LTCF, no resident was colonized with *H. influenzae* (own unpublished data).

According to Swedish national guidelines patients with moderate pneumonia are to be treated with i.v. PcG 1-3 g × 3 (www.infektion.net). The first three residents admitted to hospital were administered PcG. Interestingly, all three experienced an initial clinical improvement despite the, from a microbiological point of view, ineffective therapy. This might be in part due to the supportive therapy, but CRP-values were also lower following three days of treatment indicating an effect on the infection. As bacterial cultures from the lower respiratory tracts were not obtained, we might have underestimated the presence of other bacterial pathogens (i.e. pneumococci) in these patients. However, a recent retrospective analysis of invasive *H. influenzae* infections suggested that patient empirically treated with PcG, had a good prognosis if the treatment was altered according to culture results, while worse outcomes were seen in patients with a definite treatment of PcG in comparison to CXM [18]. Thirteen of the 15 residents and staff that were positive for the outbreak strain were eventually treated with doxycycline. A control culture was obtained approximately three weeks later. The outbreak strain still colonized the nasopharyngeal flora in five of these individuals despite adequate doxycycline treatment. No further treatment was administered, and no further cases were seen after the actions taken by the CCDC, despite the fact that some staff and residents were still colonized with the outbreak strain.

## Conclusions

We conclude that a limited outbreak of a beta-lactamase negative NTHi ST 14 with MIC values for AMP and CTX just at the clinical breakpoints occurred and was associated with severe disease. The outbreak may have been missed if ampicillin only had been used to detect BLNARs, but these strains were easily detected with the EUCAST recommended PCG 1U disk screen. The outbreak strain was closely related to a recently acknowledged lineage of NTHI. The particular NTHi clone (ST-14) seems to be associated with severe infections and further studies are needed to elucidate the virulence mechanisms and epidemiology of this particular clone.

## Abbreviations

ACL: amoxicillin-clavulanic acid
AMP: ampicillin
CCDC: County medical officer for Communicable Disease Control
CKL: cefaklor
CRB-65: Confusion-respiratory rate-blood pressure-age >65 years
CTX: cefotaxime
CXM: cefuroxime
EUCAST: European Committee on Antibiotic Susceptibility Testing
GP: general practitioner
Hi: *Haemophilus influenzae*
LTCF: long-term care facility
MIC: Minimum Inhibitory Concentration
MLST: Multi Locus Sequence Typing
NAL: nalidixic acid
NTHi: non typeable *Haemophilus influenzae*
PBP: penicillin-binding protein
PcG: benzylpenicillin
PCR: Polymerase Chain Reaction
SXT: co-trimoxazole
TET: tetracycline
